# Validation of the Greek Version of Tinnitus Handicap Inventory

**DOI:** 10.4081/audiores.2020.244

**Published:** 2020-10-06

**Authors:** Isidora Papitsi, Dimitrios G. Balatsouras, Ioannis D. Makris, Georgios Koukoutsis, Antonios Kaberos, Chara Tzavara, Thomas Nikolopoulos, Pavlos Sarafis

**Affiliations:** 1 ENT Department, “Tzaneio” General Hospital of Piraeus, 18536 Piraeus, Greece; isipapitsi@hotmail.com (I.P.); dbalats@hotmail.com (D.G.B.); kookmail@otenet.gr (G.K.); akaberos@hotmail.com (A.K.); 2 Plastic and Reconstructive Surgery Department, KAT Hospital, 14561 Kifisia, Greece; john.makris@hotmail.com; 3 National and Kapodistrian University of Athens, 11527 Athens, Greece; htzavara@med.uoa.gr; 4 Second Otolaryngology Department, School of Medicine, National and Kapodistrian University of Athens, “Attikon” University Hospital, 12462 Athens, Greece; nikolop@med.uoa.gr; 5 Department of Nursing, School of Health Sciences, Cyprus University of Technology, 3036 Limassol, Cyprus

**Keywords:** tinnitus, questionnaire, anxiety, quality of life

## Abstract

Purpose: The purpose of this study was to validate the Greek version of the Tinnitus Handicap Inventory. Method: Eighty-six adult patients with chronic tinnitus participated in the study. Sociodemographic data and medical history were recorded during the interview. The patients underwent audiological examination and they were asked to fill in three questionnaires: the Greek version of the THI (THI-GR), the Greek version of the State-Trait Anxiety Inventory (STAI) and the brief Tinnitus Severity Scale Questionnaire (TSSQ). Results: The THI-GR showed good internal consistency, comparable to the original version. Cronbach’s alpha was equal to 0.92, which suggests a robust reliability. All THI-GR subscales along with total score were significantly and positively correlated with the TSSQ grade and the audiogram results indicating the existence of convergent validity. Furthermore, THI-GR’s subscales were significantly correlated with both State and Trait subscales, which indicates a correlation between tinnitus and stress. Conclusions: This study highlighted the high reliability and validity of the THI-GR as a self-report measure for the evaluation of tinnitus-related annoyance and psychological distress in clinical practice.

## 1. Introduction

Tinnitus is the perception of sound without an external stimulus [1]. It is a common auditory symptom that can severely impair patients’ quality of life. Sufferers frequently complain about increased stress, insomnia, emotional disorders, difficulties in concentration and depression. Questionnaires are the ideal tools for quantifying tinnitus impact on everyday life, because none of the clinical or audiometric tests can estimate the severity of this symptom. As a result, measuring Health Related Quality of Life (HRQoL) in patients suffering from tinnitus should consist an integral part of the clinical approach. The identification of patients in need for further therapeutic intervention is of major importance. All these reasons highlight the need for validated and reliable instruments in the Greek clinical practice and consist the purposes of this study.

The Tinnitus Handicap Inventory (THI) is the most standardized tinnitus handicap measuring instrument in the literature. This questionnaire has been translated into many languages and has adequate reliability and validity [2].

It can be used as a robust tool to measure tinnitus distress, for the identification of patients in need of specific treatment and as a “unifactorial” scale [3].

## 2. Materials and Methods

### 2.1. Ethical Considerations

The study was performed in the outpatient audiology department of Tzaneio General Hospital. It was given written permission by the Scientific Board- Ethics Committee of the Hospital.

All the participants after being informed for the aim of this study and being ensured for the anonymity, gave consent before filling in the questionnaires. Written permission was given from the author of the original version of the THI Dr Craig Newman and for the TSSQ Dr Berthold Langguth. State-Trait Anxiety Inventory was reproduced with the permission of the publisher MIND GARDEN Inc., Redwood City, CA, USA.

### 2.2. Linguistic Validation

The original version of the THI was translated into Greek by three bilingual translators at the first phase. The translated version was then back translated into English by three other bilingual translators. The final version of the THI-GR was obtained after having reviewed these versions and corrected all the linguistic faults by a philologist. The Greek version of the THI was afterwards administered to a total sample of 20 people (10 non-tinnitus sufferers’ and 10 tinnitus sufferers’), native speakers of the Greek language, in order to crosscheck the understanding and coherence of its 25 items.

### 2.3. Participants

The research was conducted in the outpatient audiology department of Tzaneio General Hospital from February 2018 until June 2018. Patients who attended the hospital’s outpatient audiology department complaining mainly about tinnitus were informed for the objectives of the research and its voluntary character and decided whether to participate or not. The physician gathered from each patient information from the medical history and demographic data. Physical examination and audiometric tests were then performed and afterwards, each patient was asked to fill in the questionnaires. The physician gave the instructions for this procedure and was available for further questions or explanations.

Participants were 48 men and 38 women (N = 86) with mean age 60.2 years (SD = 13.8 years). Sample characteristics are presented in Table 1. 23.3% of the sample was smokers and 14% had family history of tinnitus. The duration of tinnitus had mean equal to 5.9 years (SD = 10.1) and median equal to 2 years. Most patients with either bilateral hearing loss or normal hearing reported bilateral tinnitus. Furthermore, most patients with unilateral hearing loss reported tinnitus on the same ear. 26.7% of the participants had received previous therapy, 18.6% suffered from vertigo and 25.6% suffered from headaches.

### 2.4. Questionnaires

#### 2.4.1. Tinnitus Handicap Inventory

The THI consists of a total of 25 questions which are further categorized into three subscales. The Functional subscale (11 items) estimates the impact in the areas of mental, social/occupational and physical functioning. The Emotional subscale (9 items) represents affective responses to tinnitus e.g., anger, frustration, irritability and depression. The Catastrophic subscale (5 items) is appropriate for finding out patients’ desperation, lack of control and inability to cope with their problem [4]. Three responses are available for each question (Yes: awarded 4 points, Sometimes: awarded 2 points, No: awarded 0 points). The total score ranges from zero to 100, with higher scores indicating greater perceived handicap [4].

#### 2.4.2. State-Trate Anxiety Inventory

The State-Trate Anxiety Inventory (STAI) by Charles D. Spielberger is a very popular and widely used clinical instrument for the self-assessment of anxiety. For the needs of the current survey, the Greek validated version of the STAI questionnaire was selected as an additional general tool for the measurement of anxiety in the participants. The STAI questionnaire consists of two different forms of 20 items each. The State form measures the status of the emotional life of the participant at the moment of the test taking and the Trait form draws the general anxiety profile of the participant [5]. For both forms translated into Greek the Cronbach’s alpha was found high (0.93 for the State form and 0.92 for the Trait form), indicating good reliability [6]. The score for each subtest ranges from 20 to 80 and higher scores indicate higher levels of anxiety [7].

#### 2.4.3. Tinnitus Severity Scale Questionnaire

Tinnitus Severity Scale is a short self-report scale which consists of 3 questions and can be easily administered to patients suffering from tinnitus as a first ‘screening’ tool for the tinnitus’ consequences in patients’ quality of life in a busy medical office. Two responses are available for each item (Yes/No) and the grades of tinnitus severity range from 1 (No impairment) to 4 (Severe impairment and severe disturbances in private and working life, unable to work) [8].

### 2.5. Statistical Analysis

Continuous variables are presented with mean and standard deviation (SD) and/or with median and interquartile range (IQR). Qualitative variables are presented with absolute and relative frequencies. Intraclass correlation coefficients (ICCs) were computed to evaluate test-retest reliability for all THI’s subscales. A confirmatory factor analysis (CFA) with maximum likelihood procedure was performed in order to evaluate construct validity of the THI-GR questionnaire. The variance of the latent constructs was fixed at one during parameter estimation. The fit of the CFA model was assessed using the comparative fit index (CFI), the goodness of fit index (GFI) and the root mean square error of approximation (RMSEA) [9]. For the CFI and GFI indices, values close to or greater than 0.95 are taken to reflect a good fit to the data [10]. RMSEA values of less than 0.05 indicate a good fit and values as high as 0.08 indicate a reasonable fit [10]. The internal consistency of the questionnaire was analyzed with Cronbach’s α. Reliability equal to or greater than 0.70 was considered acceptable. Spearman correlations coefficients were used to explore the association among the THI-GR subscales and the association between the THI-GR subscales and STAI subscales. Correlation coefficient between 0.1 and 0.3 were considered low, between 0.31 and 0.5 moderate and those over 0.5 were considered high. The THI-GR’s subscales were compared according to sex using Mann - Whitney tests. Also, Spearman correlations coefficients were computed for the association of age, grade (from the TSSQ) and audiogram results with the THI-GR subscales. P values reported are two-tailed. Statistical significant level was set at 0.05 and analysis was conducted using SPSS and AMOS (SPSS, Chicago, IL, USA) Statistical Software.

## 3. Results

### 3.1. Reliability

Corrected item-total correlations and Cronbach’s a if an item was deleted per factor are presented in Table 2. All corrected item-total correlations were high and internal consistency reliability was accepted with Cronbach’s alpha equal to 0.84 for Functional, 0.84 for Emotional and 0.73 for Catastrophic. Cronbach’s alpha for the total questionnaire was equal to 0.92.

Test-retest reliability was estimated with a sample of 30 patients using intraclass correlations coefficients (ICCs). All coefficients were significant at *p* < 0.001 and were equal to 0.83 for Functional, 0.80 for Emotional and 0.85 for Catastrophic, indicating acceptable stability.

### 3.2. Construct Validity

A CFA was conducted to estimate if the model fitted the data well. The CFA indicated an adequate fit of the three-factor model (RMSEA = 0.079, CFI = 0.970 and GFI = 0.960). None of the item cross loadings exceeded the item loadings on the intended latent construct.

The intercorrelations of the THI-GR subscales are shown in Table 3. All subscales were significantly and positively correlated each other and the correlations were all high.

**Figure 1:**
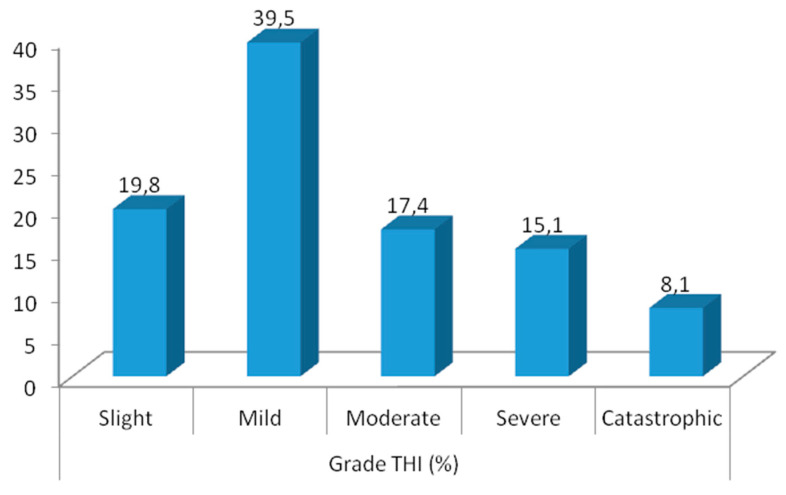
Tinnitus severity scale according to THI total score.

Sample was divided according to THI-GR total score cut off points (Figure 1). 19.8% were characterized with slight problems, 39.5% with mild, 17.4% with moderate, 15.1% with severe and 8.1% with catastrophic.

### 3.3. Convergent Validity

Correlations of THI-GR subscales with the grade from the TSSQ and audiogram results are shown in Table 4. All THI-GR subscales along with total score were significantly and positively correlated with grade and audiogram results indicating the existence of convergent validity.

**Figure 2:**
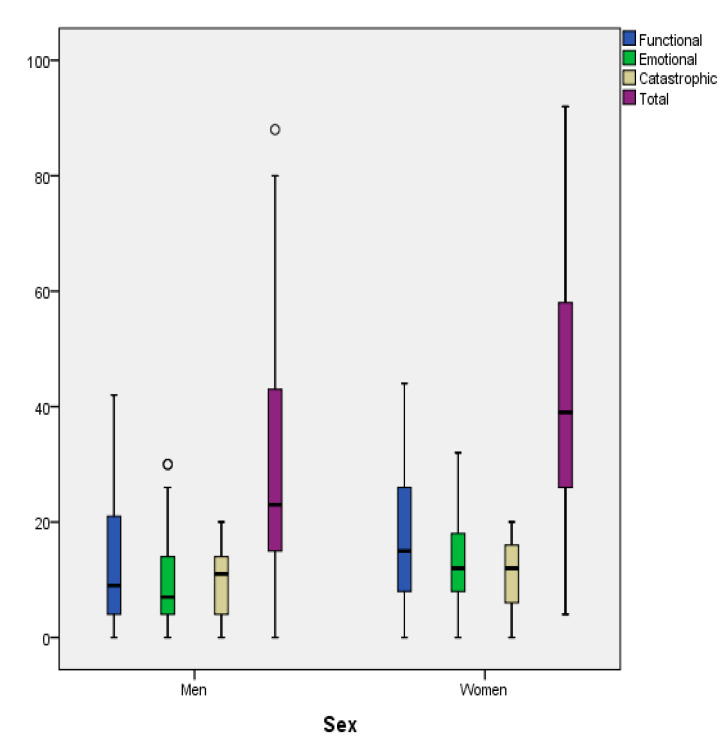
Box-plots for THI subscales according to sex.

No significant association of THI-GR’s subscales with age was found (*p* > 0.05 for all correlations). Significantly greater values were found in women as compared to men for Functional (*p* = 0.043), Emotional (*p* = 0.003) and the Total score (*p* = 0.014), indicating that women had more problems associated with tinnitus in comparison with men (Figure 2). Furthermore, THI-GR’s subscales were significantly correlated with both State and Trait subscales (Table 5).

## 4. Discussion

Quality of Life is a multi-dimensional concept which is used to measure the impact of chronic situations like illness and treatments in the patients’ daily routine. Questionnaires are the most popular Quality of Life evaluation tools that focus on informing patients about their health status and making them increasingly active in the decision-making procedure, taking into account their preferences and needs during the therapeutic interventions’ planning. The main purpose of this study was to obtain a validated measure for the evaluation of tinnitus consequences.

### 4.1. Synopsis of Key/New Findings

The results of this study demonstrated that the THI-GR has an excellent internal consistency reliability with a Cronbach’s alpha = 0.92, similar to the one found from the authors in the original version (Cronbach’s alpha = 0.93) [11]. Test-retest reliability was assessed using intraclass correlations coefficients (ICCs) in 30 participants in a second appointment and was found strong.

Significant greater values in the THI-GR score were found in women as compared to men, a fact that had not been described before in the literature [11,12,13,14,15]. Women’s quality of life was proven to be more affected from problems associated with tinnitus in comparison with men, especially in the functional and emotional area.

No significant association of the THI-GR’s subscales with age was found in accordance with the findings of other studies. [11,12,13,14,15].

The THI-GR’s subscales were correlated with both State and Trait subscales, which indicates a correlation between tinnitus and stress, a finding that differs from the study of the validation of the Russian THI [16]. In contrast, the Italian and the Chinese Cantonese versions showed a strong correlation with anxiety [12,17].

### 4.2. Strengths of the Study

The THI-GR was shown a very good Cronbach’s alpha, a fact that brings out the utility of this questionnaire as a brief and valid self-assessment tool of the perceived tinnitus handicap. This is the second time a tinnitus-specific evaluation questionnaire has been translated and validated into Greek. Panagiotopoulos et al. in 2015 validated the Mini Tinnitus Questionnaire, a 12-items questionnaire for the assessment of tinnitus’ sufferers. [18] The THI has been internationally used and adopted since its development in 1996 [4].

### 4.3. Limitations

The sample size may consist a limitation of the current study. Furthermore, all the participants who were addressed to the audiology department of the hospital came from a small region and this fact may have affected the results of the study because of the lack of heterogeneity.

### 4.4. Clinical Applicability of the Study

The study was designed in order to validate the Greek version of the THI. It is a common knowledge that the THI is a very useful and reliable tool for the evaluation of patients suffering from tinnitus. This self-report tool is a brief and easily administered questionnaire and can assess domains like physical function, emotional status and catastrophic consequences and classify the patients that are most in need for further intervention. It can also be used as a self-assessment tool for the follow up of tinnitus’ patients in the daily practice. The validated questionnaire can be very helpful in the hands of clinicians and researchers in order to quantify tinnitus self-perceived disability.

## 5. Conclusions

The results of our study suggest that the THI-GR is a reliable and valid measure for the evaluation of tinnitus’ impact in sufferers’ HRQoL. The scores obtained by the THI-GR scale can be used for the assessment of tinnitus severity, as well as the identification of the most severely affected sufferers, which are in need of urgent intervention.

## Figures and Tables

**Table 1:** Sample demographics and clinical characteristics.

Variable	N (%)
Age, mean (SD)	60.2(13.8)
Sex	
Men	48(55.8)
Women	38(44.2)
Smoking	
No	66(76.7)
Yes	20(23.3)
Occupational Noise Exposure	
No	76(88.4)
Yes	10(11.6)
Family history of Tinnitus	
No	74(86)
Yes	12(14)
Diagnosis	
Unilateral	33(38.4)
Bilateral	53(61.6)
Duration of Tinnitus (years), mean (SD)	5.9(10.1)
Audiogram results	
Normal hearing	16(18.6)
Slight/mild hearing loss	24(27.9)
Moderate hearing loss	29(33.7)
Severe hearing loss	17(19.8)
Imaging	
No	52(60.5)
Yes	34(39.5)
Previous therapy for Tinnitus	
No	63(73.3)
Yes	23(26.7)
Vertigo	
No	70(81.4)
Yes	16(18.6)
Headache	
No	64(74.4)
Yes	22(25.6)
Neck pain	
No	62(72.1)
Yes	24(27.9)
Temporomandibular Joint Disorder	
No	82(95.3)
Yes	4(4.7)

**Table 2:** Corrected Item-Total Correlations, internal consistency reliability and means of the THI subscales.

Item	Corrected Item-Total Correlation	Cronbach’s Alpha if Item Deleted	Cronbach’s a	Mean (SD)	Median (IQR)
Functional			0.84	15.3 (11.5)	12 (6–24)
12	0.69	0.82			
14	0.68	0.81			
18	0.64	0.82			
15	0.40	0.84			
1	0.52	0.83			
4	0.53	0.83			
20	0.54	0.83			
24	0.26	0.85			
2	0.40	0.84			
9	0.56	0.83			
13	0.64	0.82			
7	0.49	0.85			
Emotional			0.84	11.4 (8.8)	8 (4–16)
6	0.39	0.86			
10	0.65	0.82			
16	0.58	0.82			
22	0.61	0.82			
3	0.55	0.83			
21	0.76	0.80			
25	0.54	0.83			
17	0.64	0.82			
Catastrophic			0.73	10.1 (5.8)	12 (6–16)
5	0.36	0.73			
8	0.62	0.63			
11	0.44	0.73			
19	0.59	0.64			
23	0.64	0.62			
Total			0.92	36.7 (23.4)	29 (20–54)

**Table 3:** Intercorrelations of THI subscales.

		Emotional	Catastrophic	Total
Functional	r	0.74	0.56	0.91
	p	<0.001	<0.001	<0.001
Emotional	r		0.61	0.89
	p		<0.001	<0.001
Catastrophic	r			0.79
	p			<0.001

**Table 4:** Correlations of THI subscales with grade and audiogram results.

		Grade (TSSQ)	Audiogram Results
Functional	R	0.70	0.33
	P	<0.001	0.002
Emotional	R	0.58	0.27
	P	<0.001	0.013
Catastrophic	R	0.51	0.24
	P	<0.001	0.028
Total	R	0.70	0.32
	P	<0.001	0.003

**Table 5:** Correlations of THI subscales with State and Trait subscales.

		State	Trait
Functional	R	0.46	0.59
	P	<0.001	<0.001
Emotional	R	0.52	0.49
	P	<0.001	<0.001
Catastrophic	R	0.39	0.41
	P	<0.001	<0.001
Total	R	0.51	0.58
	P	<0.001	<0.001

## Appendix A. The THI-GR Questionnaire

1	Σας εμποδίζουν οι εμβοές σας στο να συγκεντρώνετε την προσοχή σας;	Ναι	Μερικές φορές	Όχι
2	Είναι οι εμβοές σας τόσο δυνατές, ώστε να σας εμποδίζουν στο να ακούτε άλλους ανθρώπους όταν μιλάνε;	Ναι	Μερικές φορές	Όχι
3	Oι εμβοές σας δημιουργούν θυμό;	Ναι	Μερικές φορές	Όχι
4	Oι εμβοές σας προκαλούν σύγχυση;	Ναι	Μερικές φορές	Όχι
5	Oι εμβοές σας προκαλούν απογοήτευση;	Ναι	Μερικές φορές	Όχι
6	Παραπονείστε συνεχώς για τις εμβοές σας;	Ναι	Μερικές φορές	Όχι
7	Σας εμποδίζουν οι εμβοές στο να κοιμάστε το βράδυ;	Ναι	Μερικές φορές	Όχι
8	Aισθάνεστε ότι δεν μπορείτε ποτέ να απαλλαγείτε από τις εμβοές;	Ναι	Μερικές φορές	Όχι
9	Oι εμβοές σας εμποδίζουν στο να ευχαριστείστε από τις κοινωνικές σας δραστηριότητες, όπως στο να βγείτε για φαγητό ή να δείτε μια ταινία;	Ναι	Μερικές φορές	Όχι
10	Oι εμβοές σας προκαλούν εκνευρισμό;	Ναι	Μερικές φορές	Όχι
11	Λόγω των εμβοών σας, πιστεύετε ότι πάσχετε από μια φοβερή ασθένεια;	Ναι	Μερικές φορές	Όχι
12	Σας προκαλούν οι εμβοές δυσκολία στο να ευχαριστιέστε την ζωή;	Ναι	Μερικές φορές	Όχι
13	Επηρεάζουν οι εμβοές σας την δουλειά σας ή τις οικογενειακές σας υποχρεώσεις;	Ναι	Μερικές φορές	Όχι
14	Λόγω των εμβοών σας, πιστεύετε ότι είστε συχνά ευερέθιστος;	Ναι	Μερικές φορές	Όχι
15	Λόγω των εμβοών σας, βρίσκετε δυσκολία στο να διαβάσετε ένα βιβλίο;	Ναι	Μερικές φορές	Όχι
16	Oι εμβοές σας αναστατώνουν;	Ναι	Μερικές φορές	Όχι
17	Oι εμβοές σας επηρεάζουν αρνητικά τις σχέσεις σας με μέλη της οικογένειάς σας ή φίλους;	Ναι	Μερικές φορές	Όχι
18	Βρίσκετε δύσκολο να εστιάσετε την προσοχή σας σε άλλα πράγματα, εκτός από τις εμβοές σας;	Ναι	Μερικές φορές	Όχι
19	Aισθάνεστε ότι δεν μπορείτε να ελέγξετε τις εμβοές σας;	Ναι	Μερικές φορές	Όχι
20	Aισθάνεστε συχνά κούραση, λόγω των εμβοών σας;	Ναι	Μερικές φορές	Όχι
21	Aισθάνεστε κατάθλιψη, λόγω των εμβοών σας;	Ναι	Μερικές φορές	Όχι
22	Aισθάνεστε άγχος, λόγω των εμβοών σας;	Ναι	Μερικές φορές	Όχι
23	Aισθάνεστε ότι δεν μπορείτε πλέον να αντιμετωπίσετε το πρόβλημα των εμβοών σας;	Ναι	Μερικές φορές	Όχι
24	Oι εμβοές σας επιδεινώνονται, όταν είστε σε κατάσταση στρες;	Ναι	Μερικές φορές	Όχι
25	Σας προκαλούν οι εμβοές ανασφάλεια;	Ναι	Μερικές φορές	Όχι
